# Safe laparoscopic appendectomy in pregnant patient during active labor

**DOI:** 10.1093/jscr/rjab127

**Published:** 2021-05-17

**Authors:** Charlotte S Austin, Michael Jaronczyk

**Affiliations:** Department of Surgery, Monmouth Medical Center, Long Branch, NJ 07740, USA; Department of Surgery, Monmouth Medical Center, Long Branch, NJ 07740, USA

## Abstract

Appendectomy is the standard of care in pregnant patients with acute appendicitis. The use of laparoscopy in pregnant patients with acute appendicitis is still debated, especially for patients in their third trimester. We present a case of a patient who safely underwent a laparoscopic appendectomy during early labor and subsequently delivered a healthy baby. In the correct situations and hands, laparoscopy can likely be safely used throughout pregnancy.

## INTRODUCTION

Surgical literature examining acute appendicitis in pregnancy is easily accessible. However, appendicitis when a pregnant woman is in active labor has not been reported. We report a case of a pregnant patient who underwent laparoscopic appendectomy while in labor followed by safe delivery of her baby.

Appendectomy is standard of care in pregnant patients with acute appendicitis. Studies show the rate of fetal demise from perforated appendicitis ranges from 10% to 36%, which significantly outweighs the risk of surgery and general anesthesia [1–3]. The use of laparoscopy is typically an accepted surgical approach for appendectomy during pregnancy, but the topic is still debated. Case reports and small studies assert the safety of laparoscopy in all trimesters of pregnancy, with systematic reviews showing low rates of maternal complications as well as shorter in-patient stays when compared with open appendectomy [4–7]. However, a 2018 systematic review of the topic showed a statistically significant increase in fetal loss in patients undergoing laparoscopic surgery [8].

## CASE REPORT

Our patient is a 40-year-old female at 38-week gestation who presented to Labor and Delivery with 1 day of severe right-sided abdominal pain. Her pain was sharp, constant and progressively worsening. She also noted contractions and fetal movement separate from this pain. She denied other associated symptoms, including nausea, vomiting, fevers, chills and diarrhea. There was no pain on the contralateral side and she had no history of similar symptoms, including during her first delivery. The patient felt contractions for 2 weeks that were increasing in frequency prior to presentation. Her contractions were every 2–5 minutes based on the tocometer readings. Cervical exam performed by the obstetrician revealed the cervical dilation at 1–2 cm with 50% effacement. The obstetrician diagnosed the patient with early labor. However, his concern with the separate pain led him to request surgical consultation.

**Figure 1:**
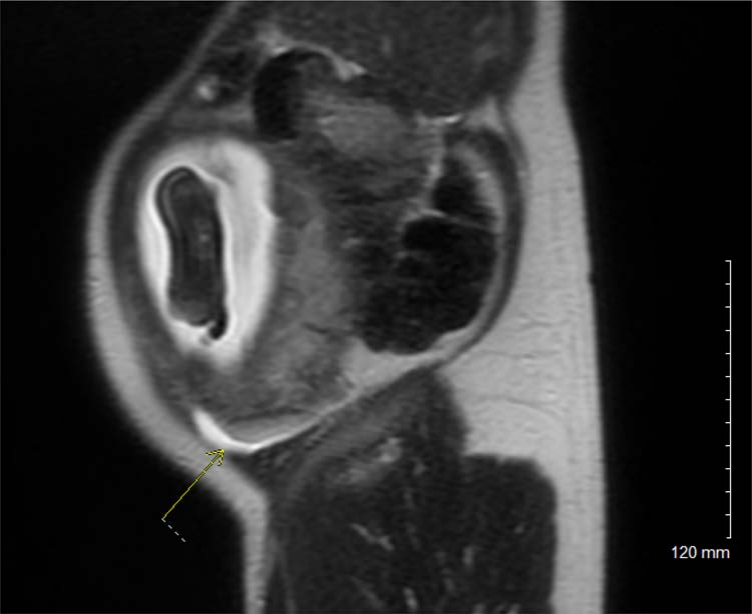
MRI pelvis, sagittal view.

**Figure 2:**
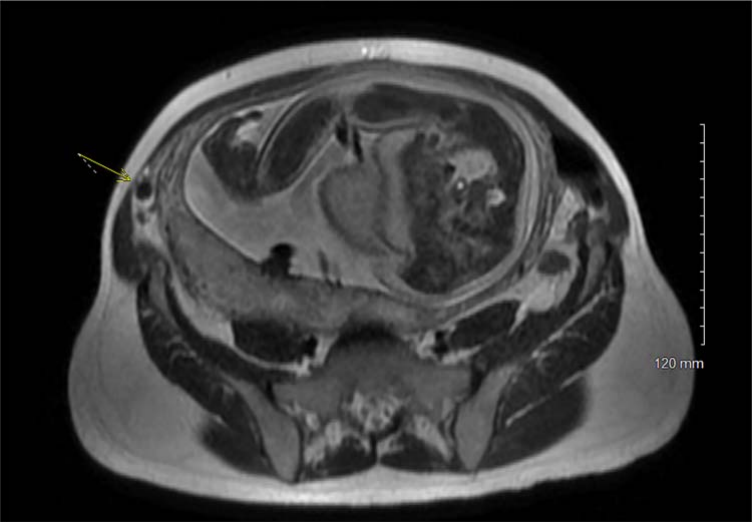
MRI pelvis, axial view.

On examination, the patient was a thin woman with a gravid uterus. She was very tender to palpation across the right lateral aspect of her abdomen, extending superiorly from the anterior superior iliac spine. Laboratory data were significant for a leukocytosis with a neutrophilic predominance. An ultrasound was obtained, but the technician was unable to visualize the appendix. The patient continued to have severe pain on the right side and an abdominal magnetic resonance imaging (MRI) was ordered. The attending radiologist reported a dilated appendiceal tip at 1 cm with surrounding inflammatory changes and free fluid, consistent with tip appendicitis (Figs 1 and 2). The cecum was noted to be superior to its normal position, with the right colon folded on itself, coursing down from the hepatic flexure before turning back upwards. The location of the appendix on the MRI correlated with the patient’s location of maximal pain and tenderness.

After discussing the case with the obstetrician, the patient and her husband, the decision was made to perform a laparoscopic appendectomy. The patient’s obstetrician was in-house and available in case of any obstetric emergency. An obstetric nurse accompanied the patient at all times. The obstetric monitors were kept in place until the surgery. She was given antibiotics preoperatively. The patient was positioned on the operating table with a bump under her right side to tilt the gravid uterus away from the pathology. After induction of general anesthesia, a Veress needle was inserted at Palmer’s point and used to insufflate the abdomen to 10 mmHg. An incision was made in the right upper quadrant and a 5-mm Optiview trocar was used to enter the abdomen with a laparoscope. There was inadequate space for visualization; therefore, the insufflation was increased to 15 mmHg. A 5-mm trocar was inserted in the epigastrium. An additional 5-mm trocar was inserted in the right anterior axillary line. The laparoscope was placed through the epigastric trocar. The appendix was identified and adhesions to the structure were dissected. The right upper quadrant trocar was upsized to 10 mm to accommodate the laparoscopic stapler. The appendix and mesoappendix were divided using a linear powered laparoscopic stapler with a white staple load. The fascia at the 10-mm trocar site was closed using a laparoscopic suture passer with a Vicryl suture.

After the procedure, the obstetric monitoring was resumed by the obstetric nurse in the Post-Anesthesia Care Unit. After recovery, the patient was brought back to Labor and Delivery for observation. Her labor continued to progress as expected. Ultimately, she delivered her daughter the following day via normal spontaneous vaginal delivery. The birth was uneventful. She was discharged home with her baby on the day following her delivery. After the discharge, the patient and the baby did not have any events or complications.

## DISCUSSION

Laparoscopic appendectomy can be safely performed in a pregnant patient in active labor. The typical approach to incisions will be different from a standard laparoscopic appendectomy. Additionally, flexibility is required to compensate for anatomic variations due to a gravid uterus. Preoperative imaging is instrumental in deciding the location for your laparoscopic incisions. Moreover, surgeon comfort and expertise must be assessed prior to any intervention and an interdisciplinary approach to complex surgical cases is essential.

## CONFLICT OF INTEREST STATEMENT

None declared.
